# Retrospective Analysis of Potential Lyme Disease Clinical Cases in Argentina

**DOI:** 10.3390/microorganisms12071374

**Published:** 2024-07-05

**Authors:** Nestor Oscar Stanchi, Dolores Oliva, Ana Vanina Lucca, Sandra Nuñez, Giuliana López, Beatriz Del Curto, María Belén Pucheta, Teresita Rigonatto, Graciela Sánchez, Serena Bonin, Giusto Trevisan

**Affiliations:** 1 Faculty of Veterinary Science, National University of La Plata, La Plata 1900, Argentina; doliva@fcv.unlp.edu.ar (D.O.); bdelcurto@fcv.unlp.edu.ar (B.D.C.); 2 Faculty of Veterinary Science, National University of Chaco Austral, Roque Sáenz Peña 3700, Argentina; vanilucca039@gmail.com (A.V.L.); sandran@uncaus.edu.ar (S.N.); trigonatto@vet.unne.edu.ar (T.R.);; 3 Faculty of Veterinary Science, National University of Noreste, Corrientes 3400, Argentina; 4 Department of Medical Sciences, University of Trieste, 34149 Trieste, Italy; sbonin@units.it (S.B.); trevisan@units.it (G.T.)

**Keywords:** Argentina, *Borrelia burgdorferi*, Lyme disease, ticks

## Abstract

Lyme disease, a multisystemic infectious disorder caused by pathogenic spirochetes of the genus *Borrelia* transmitted by the bite of ticks, typically from the family Ixodidae, pose a significant public health issue worldwide. The *Borrelia burgdorferi sensu lato* (s.l.) group encompasses the *Borrelia* Lyme Group (LG), *Borrelia* Echidna-Reptile Group (REPG), and *Borrelia* Relapsing Fever Group (RFG), with some species remaining unclassified due to culturing challenges. Research into *B. burgdorferi* s.l. infection (Lyme Group) has intensified, focusing on its epidemiology, diagnosis, and treatment. Originally identified in North America and Europe, Lyme disease has now become a global concern, with Latin American countries reporting the microorganism, the disease, and/or its vectors. In Argentina, the presence of *B. burgdorferi* and Lyme disease has sparked significant scientific and medical debate. Ecological changes due to climate and habitat shifts have expanded the geographical distribution of these ticks. Argentina, with its diverse geography and climate, hosts various tick species that could potentially act as Lyme disease vectors, raising important public health questions. The confirmed presence of *B. burgdorferi* s.l. and Lyme disease in Argentina remains contentious but relevant, necessitating thorough scientific and medical examination. This work aims to enhance understanding and discussion of Lyme disease in Argentina by presenting clinical cases and their laboratory analyses, highlighting the disease’s presence and implications in the country. Through documenting suspected clinical cases and analyzing available data on *B. burgdorferi* and Lyme disease in Argentina, this study seeks to contribute to the understanding of the disease’s current status and inform future research, prevention, and control strategies in the region. The goal is to provide a basis for addressing Lyme disease’s public health impact in Argentina and promote further investigation into this evolving issue.

## 1. Introduction

Lyme disease is a multisystemic infectious disorder caused by the bite of ticks, usually from the Ixodidae family, that are infected with pathogenic spirochetes of the genus *Borrelia*. These ticks serve as vectors in the transmission of *Borrelia burgdorferi sensu lato* (s.l.) to humans and other hosts, posing a public health problem in several regions worldwide [1].

*Borrelia burgdorferi* s.l. infections encompass three groups: the *Borrelia* Lyme Group (LG), the *Borrelia* Echidna-Reptile Group (REPG), and the *Borrelia* Relapsing Fever Group (RFG). Additionally, some Borrelia species remain unclassified due to the challenges in culturing them [2,3].

*B. burgdorferi* s.l. infection (Lyme Group) has spurred significant research efforts aimed at understanding its epidemiology, diagnosis, and treatment. While initially identified in areas of North America and Europe, Lyme disease has transcended geographical boundaries, especially in the Northern Hemisphere, becoming a global challenge. Most Latin American countries have reported the presence of the microorganism, the disease, and/or its vectors through various methodologies [1]. In the Argentine context, the presence of this disease and its causative agent, *B. burgdorferi*, has been the subject of study and debate within the scientific and medical community [4,5].

As these ticks find new ecological niches due to climate and habitat changes, the geographical distribution of the disease has significantly expanded [6]. In Argentina, a nation with vast geographical and climatic diversity, the presence of different tick species and their potential to act as vectors of Lyme disease [7] pose significant questions. Not only do climate change and habitat factors affect the emergence of borreliosis, but, perhaps more importantly, the inadequate study of this disease in the region, not just in Argentina but throughout Latin America, plays a significant role.

The presence of *B. burgdorferi* s.l. and Lyme disease in this country constitutes a relevant and evolving issue that requires comprehensive scientific and medical attention. This work aims to contribute to understanding and promoting discussion on its presence, impact, and the necessary measures to address this issue in the Argentine context.

While documented but unconfirmed cases of Lyme disease have been reported in various regions of Argentina, it is crucial to understand the dynamics of this disease and its potential to affect public health. By presenting suspected clinical cases of this disease in patients, we aim to contribute to a better understanding of the current situation of Lyme disease in the country and provide a basis for future research and prevention and control strategies.

The objective of this work is to present clinical cases and their corresponding laboratory analyses and to analyze the currently available information on the presence of *B. burgdorferi* and Lyme disease in Argentina, South America.

## 2. Methodology

This study included patients with clinical signs and symptoms compatible with Lyme disease who had not traveled outside of Argentina or, if they had traveled abroad, could recall the moment of the tick bite within Argentina. In all cases, patients presented positive Western Blot (WB) results for *B. burgdorferi*. Additionally, erythema migrans alone was considered by Lantos et al. as sufficient for diagnosis [8].

### 2.1. Case Selection

The clinical cases discussed were identified through a network of referrals, facilitated by individuals familiar with Lyme disease cases within Argentina. The inclusion criteria required all patients to have a positive WB result for Lyme disease and to have not traveled abroad before the onset of the disease. If they had traveled, they needed to accurately recall the instance of tick bites in Argentina.

One included case involved a patient who had traveled but distinctly remembered multiple tick bites. No cases were included if they did not meet these criteria. There were no cases originally thought to represent Lyme disease that were ultimately not included, as all examined cases adhered strictly to the aforementioned criteria.

### 2.2. Data Collection

Interviews were conducted either in person or via video conference. Initially, patients were allowed to freely share their history and experience with the disease to make them feel comfortable in expressing their stories. Following this, a detailed questionnaire was completed, involving a thorough anamnesis from the moment of the tick bite, including geographical location and clinical signs and symptoms from the onset to the present (noting any sequelae).

### 2.3. Verification of WB Results

For all patients, it was ensured that they provided a photograph of the WB test results (unpublished images) showing the technique used and the laboratory that conducted the test.

## 3. Description of Clinical Cases

**Case 1:** A 41-year-old female patient, residing in the city of La Plata, Argentina, who never traveled outside Argentina prior to the onset of symptoms. She had been bitten by a tick in 2017. Symptoms began a few weeks after being bitten by the tick. She found the tick, describing it as small, about 4 mm in length and dark and, at the time, dismissed it without giving it much importance; she did not present erythema migrans. During the acute phase, she experienced arthralgia, neuropathy, myositis, prolonged headache, chronic fatigue, psychosocial symptoms, cognitive dysfunction with memory loss, sleep disturbances, autonomic system alteration with pallor and vertigo, motor incoordination, and intestinal irritation. Among the other signs, she also manifested clinical signs resembling influenza with fever. Additionally, she experienced excessive weight loss, anorexia, speech disorders, location, and temporal disorders. During the process, she consulted different professionals, receiving varied diagnoses.

A clinician in the country who had lived in the USA and had knowledge of Lyme disease suspected it as a possible etiology. The patient had not previously traveled out of the country; therefore, in 2019, she traveled to Mexico, where she underwent various studies including enzyme-linked immunosorbent assay (ELISA) and western blot, both of which were positive for *B. burgdorferi*. Concurrently, polymerase chain reaction (PCR) studies were performed for the following microorganisms, all of which were negative: for *Ehrlichia* sp., *Anaplasma* sp., *Babesia* sp., and *Bartonella* spp. PCR testing for co-infections generally has a very low yield, especially outside the acute phase of the infection. While serological tests also have limitations, they may still offer useful insights.

Over the past few years, she has received various antibiotic treatments, with temporary improvements and numerous relapses, with neurological symptoms remaining that hinder her normal social functioning.

Laboratory Diagnostic Tests: her blood tested positive for IgG both ELISA and Western Blot (VlsE band): reference value: positive > 1100. Result: *B. burgdorferi* IgG VIsE 1185 Positive. Details on proteins resulted positive in WB are reported in Table 1. In the cerebrospinal fluid the WB was positive in IgG for the VlsE band and the CXCL13 [9] was also positive.

*B. burgdorferi* IgG VIsE 1185 Positive CXCL-13 test was performed in cerebrospinal fluid (*B. burgdorferi*–neuroborreliosis). Reference value: Positive > 1100. Result: Positive.

**Case 2:** A 47-year-old female patient, residing in the city of Berisso, Argentina, who never traveled outside Argentina prior to the onset of symptoms. She was bitten several times by ticks 17 years ago (2007). She did not remember noticing the presence of erythema migrans on her body. During the first years, she presented several symptoms, including arthritis, neuropathy, neuritis, arrhythmia, muscle pain, prolonged and very intense headaches, intense physical and mental fatigue that did not improve with rest, and emotional tension lasting more than six months. Additionally, she experienced psychosocial symptoms such as manifestations similar to panic syndrome, anxiety, and social isolation. She suffered from cognitive dysfunction, which impaired her thought, language, perception, memory, concentration, reasoning, and attention. Sleep disturbances, hypersensitivity to noise and light, and autonomic disturbances such as cutaneous pallor and vertigo were also present. She experienced motor incoordination affecting her walking and speaking, as well as intestinal irritation. Neuroendocrine disorders, including rapid weight loss and anorexia, were noted. In 2016, her symptoms worsened. She was treated with penicillin, clindamycin, and doxycycline.

One year later (2017); the diagnosis was made in Mexico by ELISA and WB; both positive. Concurrently; studies were performed for the following microorganisms; all of which were negative: PCR for *Ehrlichia canis*; *Ehrlichia chaffeensis*; *Anaplasma phagocytophilum*; *Rickettsia* spp.; *Babesia microti*; and *Bartonella* spp. Currently; she suffers from neuropathy, pain, and muscle weakness. Serological tests might have been useful but were not performed.

Laboratory Diagnostic Tests: ELISA anti *B. burgdorferi* s.l. IgG VIsE (*B. burgdorferi* s.l.)/IgM (*B. burgdorferi* s.l.). Reference: Positive IgG/IgM > 1100. Result: *B. burgdorferi* IgG VIsE 1612 and *B. burgdorferi* IgM 3015.

Western Blot: Western Blot IgG/IgM anti *B. burgdorferi* s.l. Positive bands: OspC, p41, VIsE, and VIsE *B. burgdorferi* s.l. Reference: Specific antigenic bands: p18, p19, p20, p21, OspC (p25), p39, p83, Lipid Bb, Lipid Ba. Result: positive.

**Case 3:** A 51-year-old male patient, residing in the city of Mendoza, Argentina. The place of infection was Las Yungas, province of Salta. This area is characterized as a reserve with transitional zones towards the Chaco park. Las Yungas are characterized by a strong altitudinal gradient that corresponds to climatic variations, associated with wide variability in the specific composition of vegetation [10]. Although the patient traveled abroad before his clinical case, he is included in the study due to his confidence regarding the timing of tick bites. The patient was bitten in 2016 by numerous ticks. Symptoms and clinical signs included severe pain and serous secretion in the knees (site of the bite), muscle pain, and marked weakness, concomitantly with a significant weight loss of 20 kg. The patient was seen by a general practitioner who observed these secretions, but they were not treated. Unfortunately, no studies were conducted on them. He also presented psychosocial problems with frequent forgetfulness and speech difficulty. He manifested an erythematous reaction in red circles that covered his entire leg, but no photographic evidence exists beyond the patient’s account. After visiting various professionals, one of them suspected Lyme disease, and ELISA and Immunoflourescent assay (IFA) studies were performed, both yielding positive results. In order to confirm the study, a serum sample was processed in Mexico by Western Blot, also yielding a positive result. The patient was treated with antibiotics (doxycycline), significantly improving his condition. Currently, he suffers from joint pain sequelae.

Laboratory Diagnostic Tests: ELISA anti *B. burgdorferi* IgG VIsE (*B. burgdorferi* s.l.)/IgM (*B. burgdorferi* s.l.). Reference: Positive IgG/IgM > 1100. Result: *B. burgdorferi* s.l. IgG VIsE 1123 and *B. burgdorferi* IgM 1001. Result: IgG positive, IgM suspicious.

Western Blot: Western Blot IgG/IgM anti-*B. burgdorferi* s.l. Positive bands: p21 BB_K53, OspC, p41, and p83 and VIsE. The presence of a positive p83/100 band is usually related to late Lyme disease manifestations [11]. Reference: Specific antigenic bands: p18, p19, p20, p21, OspC (p25), p39, p83, Lipid Bb, Lipid Ba. Result: Positive.

**Case 4:** A 43-year-old female patient, residing in Mendoza, Argentina who never traveled outside Argentina prior to the onset of symptoms. The potential site of infection was in areas near her residence, presumably at the age of 8, although she does not recall being bitten by a tick. The disease remained latent for several years, with no clinical or cutaneous manifestations. She was diagnosed with fibromyalgia. The patient had not traveled abroad before the onset of symptoms. From adolescence onward, she began experiencing rash, arthritis, neuropathy, muscular pain, and recurrent erythema. Worsening clinical signs and symptoms, eventually rendering her bedridden. Laboratory studies conducted in USA revealed positive IFA for IgG, a result confirmed through multiple repetitions of the procedure. Finally, a western blot was performed in USA, interpreted as positive. Following a four-month course of treatment with doxycycline, the patient exhibited significant improvement without any lingering sequelae.

Laboratory Diagnostic Tests: Western Blot: IgG Western Blot. Reference: Bands 31 and 34 are typically present in Lyme-vaccinated patients. Anti-viral antibodies may cross-react with the 93 kDa antigen. A sample is considered positive if two or more bands are present (bands 23, 31, 34, 39, 41, and 93). Result: Bands 31 and 41 weakly positive on the automated reader, reported as reactive upon visual inspection. Result: Positive.

**Figure 1:**
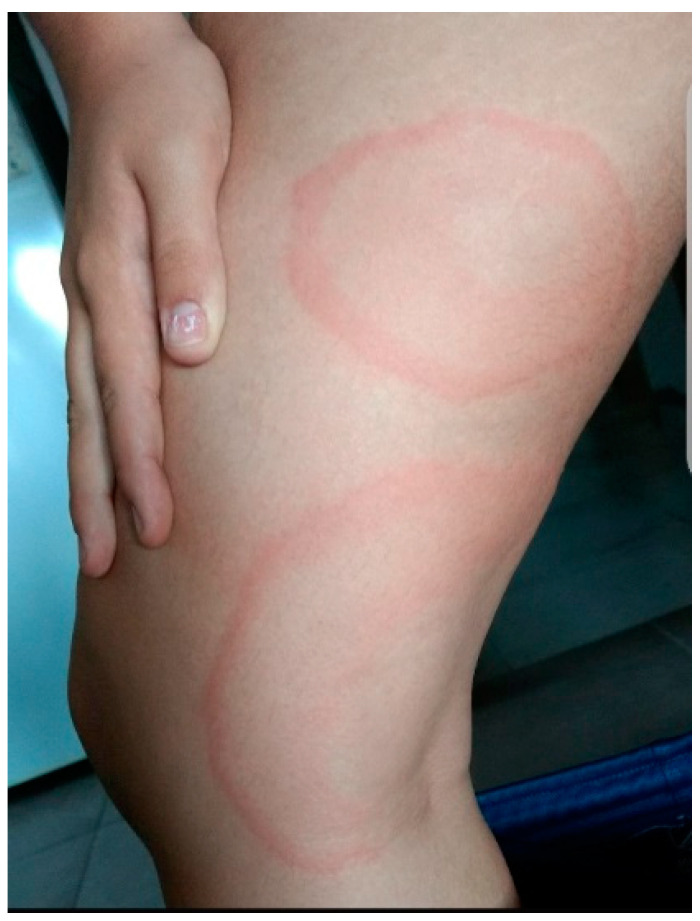
Erythema migrans in case 5. The erythema migrans is only on the knee, which appeared first, with the tick bite mark visible at its center. The other lesion (above) is a secondary annular erythema that appeared later, with no signs of a tick bite.

**Case 5:** A 19-year-old female patient, residing in Mar Chiquita, Buenos Aires, Argentina, who never traveled outside Argentina prior to the onset of symptoms. The potential site of infection was in areas near her residence, presumably at the age of 12, although she does not recall being bitten by a tick. She presented with a typical erythema migrans lesion of Lyme disease (see Figure 1). Subsequently, she developed worsening signs and symptoms, including arthritis, neuropathy, disorientation, mental lapses, cramps, fatigue, and cognitive impairment, along with sleep disturbances. Upon suspicion of Lyme disease, she underwent IF testing in Argentina, yielding a positive result. Additionally, ELISA and Western Blot tests were performed in Mexico, both returning positive results. She received treatment with doxycycline and ceftriaxone, leading to complete resolution of symptoms.

Laboratory Diagnostic Tests: Immunofluorescence Assay (IFI) for anti *B. burgdorferi* s.l. IgG and IgM: Reference values negative. Result: IgM negative, IgG positive 1/80. ELISA for anti *B. burgdorferi* IgG VIsE (*B. burgdorferi*)/IgM (*B. burgdorferi* s.l.). Reference: Positive IgG/IgM > 1184. Result: *B. burgdorferi* IgG VIsE 1612 and *B. burgdorferi* s.l. IgM 3062. Result: Positive for IgM and IgG.

Western Blot: IgG/IgM Western Blot for *B. burgdorferi* s.l. Positive bands observed: p41, p83 (supporting late Lyme disease manifestations), and VIsE. Reference: Specific antigenic bands: p18, p19, p20, p21, OspC (p25), p39, p83, Lipid Bb, Lipid Ba. Result: Positive.

## 4. Analysis of Lyme Disease Patient Data

From the analysis of the data, symptoms, and clinical signs of the five patients, the following findings were obtained: all patients (100%) were found in a habitat where ticks could be found; only 2 of them (40%) recall being bitten by one, and, in both cases, they didn’t consider it significant. Additionally, three of them (60%) showed cutaneous manifestations of the disease, especially in two cases (40%), producing the typical erythema migrans. This cutaneous manifestation recurred in only one case (20%). Three of them (60%) presented signs and symptoms similar to the flu. Only one patient had facial nerve involvement (20%). The majority of the patients (4 out of 5) experienced fainting spells (80%) and severe headaches (100%). All of them suffered from joint inflammation, joint and muscular pain, and intense fatigue (5/5), and they all experienced cognitive and sleep disturbances (100%). They also had difficulty walking and speaking (40%) and had mood swings or psychiatric disorders (80%).

The results of the bands tested by Western Blot for the five Lyme disease case patients are presented in Table 1. The positivity criteria for diagnostic tests vary across different countries and laboratories, as shown in Table 2.

## 5. Discussion

Confirmatory or definitive tests for Lyme disease generally involve specific and sensitive serological tests, such as ELISA followed by western blot [11]. These tests aim to detect the presence of antibodies against *B. burgdorferi* in the patient’s blood.

Western blot is considered the gold standard for confirming Lyme disease. This test demonstrates the immune system’s reaction to specific proteins of *B. burgdorferi*, allowing for the identification of specific antibodies produced in response to the infection. The presence of specific bands on the WB, along with clinical signs and symptoms and a history of tick exposure, can confirm the diagnosis of Lyme disease [12,13]. However, positivity criteria vary depending on the proteins used in the techniques, the countries where they are developed, and the standardization and validation of the respective laboratories where the tests are performed. Considering these parameters, two of the patients would be positive for the Centers for Disease Control and Prevention (CDC) [14] and one for the European Concerted Action on Lyme Borreliosis (EUCALB) [14]; all would be positive for the laboratory that performed the test, highlighting that they were all positive for the flagellar antigen of *Borrelia burgdorferi* s.l., which does not have antigenic variation among pathological species [15]. Although serological tests, also including Western blot, cannot definitively discriminate among genospecies of *Borrelia burgdorferi* s.l., in our cohort, 3 patients reported Western blot signs specific for p83/100 of *B. afzelii*, which is very rare among Americans. In Mexico, two cases have been reported as well as a clinical case of acrodermatitis chronica atrophicans, a clinical manifestation mainly due to *B. afzelii* [16,17].

There may be strains of *B. burgdorferi* in Argentina that differ from those found in Europe and the USA. This possibility is crucial as it suggests that local variations in the pathogen could impact diagnosis, treatment, and epidemiological understanding. Research focused on identifying and characterizing these strains is essential to determining their genetic, antigenic, and clinical differences. Such studies would enhance our knowledge of Lyme disease in Argentina, potentially leading to better prevention and control measures tailored to the regional context [18].

In addition to serological tests, molecular tests like PCR can detect *B. burgdorferi* genetic material in blood, cerebrospinal fluid, or other tissues [19,20]. However, PCR may be less sensitive than serological tests and its effectiveness can depend on the disease stage and sample quality. Healthcare professional awareness of clinical symptoms and signs is crucial for timely and accurate Lyme disease diagnosis. While PCR testing for co-infections generally has a low yield, especially outside the acute phase, serological tests, despite their limitations, can still provide valuable insights.

We propose that studies should be conducted to clarify the presence and types of borrelioses in Argentina. Comprehensive surveys are needed to identify potential reservoir hosts, including both wild and domestic animals, focusing on species known to harbor *Borrelia* spirochetes. Detailed tick surveillance programs across various regions should identify tick species by collecting and identifying ticks from different environments and hosts. Molecular and serological analyses are essential to detect *Borrelia* species within ticks. Longitudinal studies should monitor the incidence and spread of Lyme disease and other borrelioses by tracking clinical cases and conducting seroprevalence studies in human and animal populations. Implementing advanced diagnostic techniques such as PCR; next-generation sequencing; and serological assays will confirm the presence of *Borrelia* spirochetes and other pathogens in collected samples. These efforts will help us understand the ecology and epidemiology of borrelioses in Argentina and develop effective prevention and control strategies [21,22].

The clinical cases presented are those that have been considered in light of information from European and U.S. clinics. However, other clinical presentations might exist and should be taken into account, such as Baggio–Yoshinari Syndrome from Brazil [23].

It is important to note that the diagnosis of Lyme disease should be based on a combination of clinical findings, laboratory tests, and a history of tick exposure, as no single test is definitive [24,25]. Nevertheless, erythema migrans is pathognomonic for Lyme Borreliosis; therefore, alone, it can be considered diagnostic, as in our case number 5 [8].

This is the first study that reveals the presence of Lyme disease in Argentina, as shown by the analysis of five cases. It is necessary to highlight this first step to raise awareness in the healthcare system.

This study underscores the importance of continued research and active surveillance of Lyme disease to better understand its epidemiology, ensure timely diagnoses, and develop effective prevention and control strategies. Emphasizing the need for heightened awareness and research efforts is essential for improving the management and prevention of Lyme disease in Argentina.

## Figures and Tables

**Table 1:** Western Blot results for different case patients.

Case.	Control	IgM	IgG	P17/18 (DbpA)	p19/(20 (OspE)	p21/p25 OspC) *B. burgdorferi s.s.*	OspC *B. garinii*	OspC *B. afzelii*	P30/P32 (OspA)	VIse *Bbss*	VIsE *B. garinii*	VIsE *B. afzelii*	p39 (BmpA)	P41 (Flagellin) *Bbsl*	p58 (HSP)	p83/p100
1	+	−	+	−	−	+	−	−	−	+	−	−	+	+	−	+
2	+	−	+	−	−	−	+	−	−	+	+	−	−	+	−	−
3	+	−	+	−	−	+	+	−	−	+	−	−	−	+	−	+
4	+	−	+	+	−	−	−	−	−	+	−	−	−	+	−	
5	+	−	+	−	−	−	−	−	−	+	−	−	−	+	-	+

(+) Positive, (−) Negative. Tests for Cases 1, 2, 3, and 5 were performed at the specialized laboratory for tick-borne diseases (BioGeneTicks, Hidalgo, Mexico). The test for Case 4 was performed at Igenex Laboratory, California, USA. Bbss = *Borrelia burgdorferi sensu stricto*, BbsL 0 *Borrelia burgdorferi* s.l.

**Table 2:** Laboratory Studies for Lyme Disease Diagnosis in patients with clinical signs and symptoms of Lyme disease.

			Western Blot		Laboratory Criteria	
Case	IFA	ELISA	Laboratory *	Positivity for *Borrelia burgdorferi sensu lato*	CDC	EUCALB
1	NP	+	+	+	+	+
2	NP	+	+	+	−	−
3	NP	+	+	+	+	−
4	+	NP	+	+	−	−
5	NP	+	+	+	−	−

* Criteria of Laboratory that performed the test; NP: Not performed, (+) Positive, (−) Negative. CDC: Centers for Disease Control and Prevention, EUCALB: European Concerted Action on Lyme Borreliosis.

## Data Availability

The original contributions presented in the study are included in the article, further inquiries can be directed to the corresponding author.
